# Sequential Combination of a Strong Interferon Inducer Viral Vector With Low Doses of Nivolumab Plus Ipilimumab Could Provide Functional Cure in Chronic Hepatitis B Virus infections: Technical Report Proposing a New Modality

**DOI:** 10.7759/cureus.22750

**Published:** 2022-03-01

**Authors:** Tibor Bakacs, Rifaat Safadi, László G Puskás, Liliána Z Fehér, Imre Kovesdi

**Affiliations:** 1 Department of Probability, Alfred Renyi Institute of Mathematics, The Eotvos Lorand Research Network (ELKH), Budapest, HUN; 2 The Liver Unit, Hadassah Medical Organization, Hadassah Hebrew University Medical Center, Jerusalem, ISR; 3 Drug Development, AVIDIN Ltd., Szeged, HUN; 4 Virology, ImiGene, Inc., Rockville, USA

**Keywords:** ipilimumab, nivolumab, off-label low dose checkpoint inhibitors, artificial viremia, attenuated viral vaccine vector, viral superinfection therapy, functional cure, chronic hepatitis b infection

## Abstract

Based on the recommendation of the International Coalition to Eliminate hepatitis B virus (ICE-HBV), we intend to mimic the spontaneous resolution of HBV infection to achieve a functional cure of chronic hepatitis B virus (HBV) infection. To this end, we propose sequential targeting of the innate and adaptive host immune responses. Long-term suppression of HBV replication and hepatitis B surface antigen (HbsAg) production will be achieved first by inducing a strong innate immune response. The clinically validated viral superinfection therapy (SIT) will be administered, which employs an attenuated, non-lytic, double-stranded RNA (dsRNA) infectious bursal disease virus (IBDV) that provides an exceptionally strong interferon (IFN) response. Then, the exhausted HBV-specific T cell function will be restored by blocking the cytotoxic T lymphocyte-associated antigen-4 (CTLA‐4) and programmed cell death protein 1 (PD‐1) receptors with immune checkpoint inhibitors (ICIs). In order to minimize any risk of toxicity, off-label low doses of nivolumab (0.5 mg/kg) plus ipilimumab (0.3 mg/kg) will be administered, the safety and efficacy of which has already been demonstrated in 131 unselected stage IV cancer patients. We predict that this combination therapy will provide sustained off-treatment virological and clinical responses during a relatively short treatment period.

## Introduction

More than 250 million individuals worldwide are chronically infected by the hepatitis B virus (HBV), which causes over 780 thousand deaths every year [1]. The standard of care consists of two main antiviral therapies: nucleos(t)ide analogs (NAs) and pegylated interferon alpha (PEG-IFN).

Even after prolonged administration, NAs are not curative because they can only suppress viral replication but not eliminate covalently closed circular DNA (cccDNA), the transcriptional template in infected hepatocytes. Treatment, therefore, must continue for life in most patients. Long‐term use of NAs is associated with toxicity, noncompliance, viral resistance, and unsustainable cost implications. Unfortunately, NA treatment does not eliminate the risk of hepatocellular carcinoma (HCC). Therefore, even virally suppressed patients may still develop liver cancer, particularly in cirrhotic livers. Thus, 80 million people may die from HCC [2].

PEG-IFN has a definitive treatment course, and responders may maintain a virologic response after drug withdrawal. Antiviral immune modulation IFN therapy has unique mechanistic advantages over NAs because it disrupts immune tolerant states in chronic hepatitis B (CHB) patients who are unable to mount an immune response to clear the infection. However, the safety and efficacy of PEG-IFN are still not satisfactory [1].

Despite a variety of antiviral agents in clinical development targeting various steps of the HBV life cycle (e.g., viral RNA interference molecules, capsid assembly blockers, immune checkpoint inhibitors (ICIs), hepatitis B surface antigen (HbsAg), cccDNA generation blocking molecules, and innate immune system modulators), none of them is expected to effectively cure CHB in the foreseeable future [1,2].

New strategy to reduce the number of productively infected cells and stimulate HBV-specific T cells

For the development of a functional HBV cure (sustained undetectable HBsAg and HBV DNA in serum with or without seroconversion, with persistently low amounts of intrahepatic cccDNA and HBV DNA integration), it is important to remember that in immunocompetent adults, over 90% of HBV infection is self-limiting [2]. In such cases, a robust HBV-specific T-cell response is directed against viral proteins. During chronic infection, however, the immune responses are weak and HBV-specific T cells display the hallmarks of T cell exhaustion. The persistence of HBsAg could explain why the HBV-specific T cells remain dysfunctional compared to those patients who clear HBsAg. Therefore, a functional HBV cure should be achievable, provided therapy addresses both the high viral burden and weak T cell immune responses, respectively [2].

Consistent with this assumption, the International Coalition to Eliminate HBV (ICE-HBV) emphasized that a complex interplay of innate and adaptive immune responses is essential for viral clearance. To achieve this goal, they proposed two strategies: (1) curing HBV infection without killing infected cells, and (2) inducing immune control to eliminate remaining infected cells [2].

The feasibility of enforcing specific immunity against HBV has been supported by previous observations [3]. An HBsAg carrier patient with active viral replication was treated for leukemia by bone marrow transplantation (BMT) from an HBV immune donor. Adoptive transfer of HBV-specific lymphocytes from the seropositive immune donor into the seronegative HBV carrier results in the elimination of HBV and seroconversion. Clearly, allogeneic BMT, with its associated high morbidity and mortality, cannot be routinely used as a therapeutic means for clearance of HBV. ICI drugs, however, are capable of restoring the function of the CHB patients’ own exhausted HBV-specific T cells [2,4].

## Technical report

Following the recommendations of ICE-HBV, we propose the combination of two clinically tested modalities for the functional cure of CHB patients. The broad-spectrum post-infection antiviral superinfection therapy (SIT) [5] will first stop HBV replication, and thus persistent exposure of T cells to high antigen loads and antigen presentation by hepatocytes. Then, in order to eliminate the remaining infected hepatocytes, the dysfunctional HBV-specific T-cell system will be restored by the sequentially administered off-label low-dose nivolumab plus ipilimumab ICI therapy [6]. This new combination therapy will hopefully induce lifelong viral control by a sustained immune response similar to that observed in acute or chronic hepatitis B resolvers.

The broad-spectrum live attenuated dsRNA IBDV vector activates the antiviral innate responses by inducing IFN-stimulated genes and other signaling cascades from within

The broad spectrum SIT has successfully treated hepatitis A virus (HAV) infection in marmoset monkeys, HBV, and hepatitis C virus (HCV) infections in 42 patients with acute and in 4 patients with chronic (parenchymally decompensated) disease with various life-threatening complications [5]. Recently, SIT was proven to be safe and effective in COVID-19 disease and in severe herpes zoster ophthalmicus (HZO) infection, respectively [7,8].

The type I IFN system is a major player in antiviral defense against all kinds of viruses. Infected cells respond by synthesizing and secreting type I IFNs (IFN-α/β) which signal the body about the presence of dangerous intruders. Haller et al. reviewed the interactions of several viruses and cells, demonstrating the power of the IFN system to eliminate pathogenic viruses [9]. It was demonstrated that HBV replication and gene expression in the liver were inhibited by the local induction of IFNs during adenovirus and murine cytomegalovirus infections. It was suggested that induction of these cytokines in the liver and other infected tissues of chronically infected patients might have therapeutic value [10].

Recently, several oncolytic viruses have been developed as promising agents for cancer therapy because they trigger immune responses that can boost anticancer immunity. One of the important systems involved is the type I IFNs that play a central role in the induction of anticancer immunity. A good example of these oncolytic viruses is the reovirus, which induces a strong IFN response before destroying tumor cells by replication and spreading through the tumor [11]. In the context of treating CHB, it is important to use a non-pathogenic virus that induces a strong IFN response without lysing the host cells. This attenuated infectious bursal disease virus (IBDV) drug candidate R90378 is one such extremely rare virus. Its dsRNA genome induces a very strong IFN response without harming liver cells [12]. Therefore, it is a perfect drug candidate for SIT therapy.

dsRNA is a molecular structure associated with viral infection because most viruses produce dsRNA at some point during their replication cycle [13]. During SIT, the non-pathogenic attenuated IBDV delivers its dsRNA cargo to host cells and activates their natural antiviral interferon gene defense system from within via Toll-like receptors (TLRs; e.g., TLR3, TLR7, TLR8, and TLR9) [5]. TLRs are a family of innate immune-recognition receptors that recognize molecular patterns associated with microbial pathogens.

The particular cytokine induction profile of IBDV separates antiviral efficacy from inflammation which improves the therapeutic index

During IBDV mass vaccination programs in poultry over the past 50 years, no cases of zoonosis were ever reported in workers of chicken coops and/or IBDV vaccine production facilities. For the regulatory authorities, however, even a very low risk of zoonosis is a justified concern. To produce batch-to-batch consistency and prevent spontaneous mutations of the IBDV drug candidate, a reverse genetics technology was used [12].

The attenuated reversely engineered IBDV serotype R903/78 strongly induces IFN-β (about 30-fold) and IFN-λ (5 to 10-fold) in the A549 human lung adenocarcinoma cell line. In contrast to other viruses (e.g., adenovirus, encephalomyocarditis virus), IFN-γ is not induced [7]. Furthermore, IBDV does not lyse the A549 cells nor any other mammalian cell lines tested, providing safety compared to the use of lytic viruses [12]. In such a way, antiviral efficacy is separated from inflammation. In this context, it is important to recall that IFN-β has the highest binding affinities for the IFN receptors, however, it is more toxic in patients, probably because of its high antiproliferative activity. In addition to type I IFN, type III IFNs, consisting of four IFN-λ subtypes, have been considered as an alternative in the treatment of CHB [1]. Therefore, the particular cytokine induction profile of IBDV maximizes antiviral action with minimal side effects in CHB patients, opening up the therapeutic window.

IBDV was used usually for 24 weeks, but up to 52 weeks in severe hepatitis cases. In this context, it is important to note that multiple oral IBDV administrations generated high levels of neutralizing antibodies in mice. Notwithstanding, effective oral IBDV administration was possible in the presence of high levels of IBDV neutralizing antibodies [12]. Important to note that data in mouse tissues indicated viral stability and genome accumulation under the multiple dosing scenario rather than viral replication. This is consistent with clinical observations in decompensated hepatitis patients when large doses of the viral preparation were administered continuously over a long period for the maintenance of ‘artificial viremia’ [5].

IBDV is not known to infect human beings naturally. However, three weeks after the last IBDV dose, the sera of the COVID-19 and HZO patients contained anti-IBDV neutralizing antibodies. These data confirmed the results obtained in animal models that following oral administration of IBDV, neutralizing antibodies were induced in patients as well [7,8].

**Figure 1:**
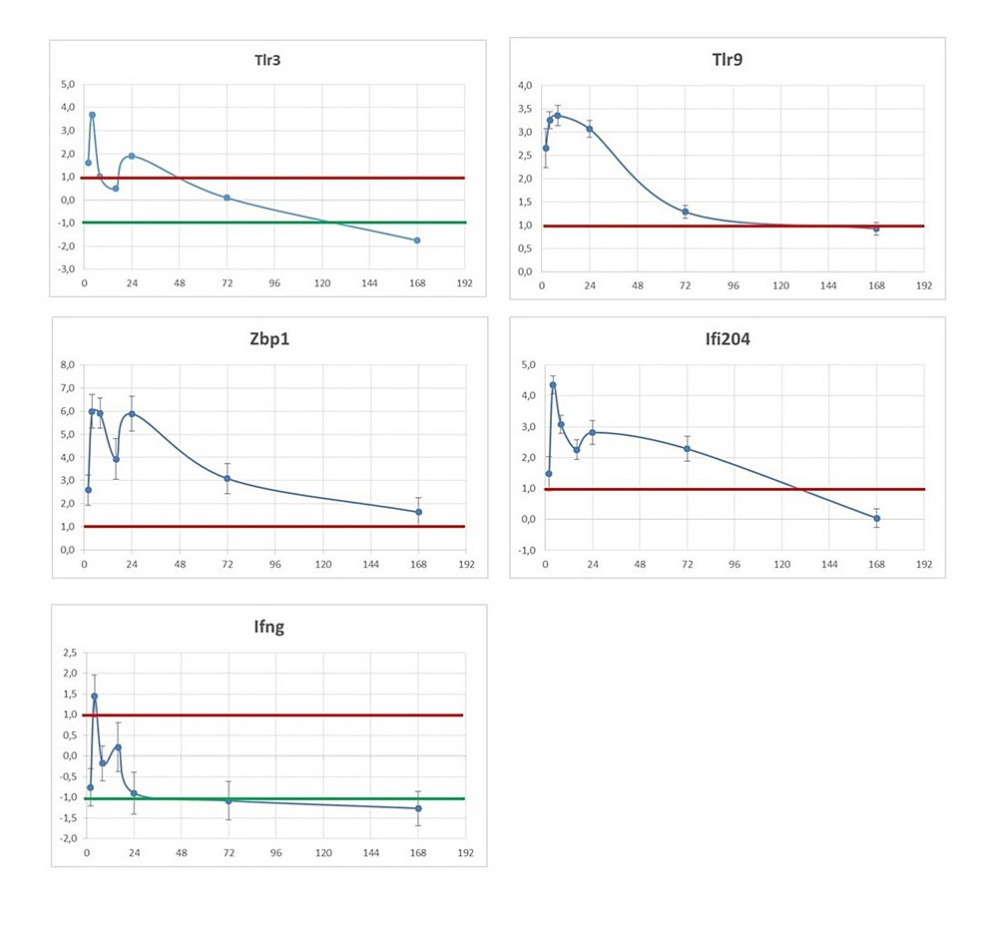
Analysis of expression levels of virus-activated genes following intravenous injection of IBDV (R903/78) drug candidate. X = delta-delta Ct values presented as log2 values; Y = time after IV injection of IBDV (R903/78) (hrs). The mean gene expression values above the red line denote increased, while the values below the green line indicate decreased gene expression levels.

Although the patients with decompensated liver disease had high-level viremia, which is one of the key drivers of cytokine storm, IBDV treatment did not induce the excessive release of pro-inflammatory cytokines. In fact, no serious side effects were observed during IBDV therapy [5]. The safety of IBDV is in stark contrast to the toxicity of systemic IFN-based therapy that causes significant morbidity, requiring dose reduction or even discontinuation of therapy [14]. A possible explanation for the very different safety of SIT and systemic IFN therapy could be that the target range of the two therapies is different. IFN-α acts on almost all human nucleated cells evoking a complex reaction pattern when administered systemically [15]. Viruses, in contrast, have very restricted cellular and host tropism. The dsRNA of IBDV is recognized by specific receptors (e.g., TLR3). This way, several gene families are activated from within the endosomes. Expression levels of IFN-related genes such as the toll-like receptor 3 (Tlr3), toll-like receptor 9 (Tlr9), Z-DNA binding protein 1 (Zbp1), interferon-activated gene 204 (Ifi204), interferon-gamma (Ifn-g) genes are increased even after a single intravenous injection of IBDV (R903/78) drug candidate (Figure 1).

Regardless of the specific mechanism of action, it is already clear that the systemic IFN-based and virus-based therapeutic modalities are different [5]. We hypothesize that the dsRNA of IBDV prevents immune surveillance evasion more effectively than systemic IFN therapy because several innate immune system gene families are induced. However, unlike RNA interference or CRISPR/Cas9 technologies [2], SIT has durable off-treatment effects while it has no off-target safety issues.

The off-label low-dose ipilimumab plus nivolumab anticancer therapy could induce proliferation of HBV specific T-cells and restore adaptive immunity

The remarkable success of ICI in patients with cancer could offer a promising new complementary strategy to reinvigorate the impaired HBV-specific T cell response. The problem with ICI therapy is that the long-lasting objective of cancer regression can only be achieved by compromising tolerance to healthy self-tissues [16]. The inconvenient truth is that autoimmunity is emerging as the nemesis of immunotherapy. While the safety and efficacy profiles of ICI agents were established in cancer patients, it remains to be seen how those parameters can be applied to CHB patients. A single dose of either 0.1 or 0.3 mg/kg of nivolumab, with or without GS-4774, was well tolerated and effective in virally suppressed hepatitis B e-antigen (HBeAg) negative patients [4]. Notwithstanding, the examination of combinatorial strategies for the treatment of CHB is encouraged. With higher dosages or combination therapy, better recovery of T cells would be achieved, but the risk of irAEs would also be increased. Therefore, it seems promising to administer low-dose ICI therapy for the reinvigoration of the impaired HBV-specific T cell response in CHB patients.

The safety and efficacy of the off-label low-dose nivolumab plus ipilimumab combination ICI therapy was demonstrated in 131 unselected stage IV solid cancer patients with 23 different histological types of cancer who had exhausted all conventional treatments [6]. The anticancer therapy consisted of hyperthermia and interleukin 2 (IL-2) treatment combined with ipilimumab (0.3 mg/kg) plus nivolumab (0.5 mg/kg). The median overall survival was 19.3 months, while the immune-related adverse events (irAEs) of the World Health Organization (WHO) Toxicity Scale grades 1, 2, 3, and 4 were observed in 23.7%, 16.0%, 6.1%, and 2.3% of patients, respectively. Based on its irAEs profile, this new combination treatment is much safer than that of the established protocols without compromising efficacy. In fact, the median overall survival of the patients treated with the low-dose ICI therapy compares favorably with the real-world outcomes for patients treated with registered doses of ICIs in the USA Veterans Affairs System [17]. We predict that this sequential combination therapy will be able to accomplish a functional cure of CHB infection during a definitive treatment course.

## Discussion

The current goal of HBV therapy is to accomplish a partial cure. However, a functional cure is achievable if not only viral replication is suppressed but a durable HBV-specific T-cell response is restored that mimics spontaneous resolution of HBV infection. To this end, modulating a single pathway may not be sufficient [2].

A proof-of-principle trial is proposed here as a model for next-generation anti-HBV therapy investigating the combination of superinfection therapy with immune-enhancing modalities (an off-label low-dose ICI combination therapy). We would like to evaluate whether such a combination can inhibit both viral replication and reinvigorate antiviral HBV-specific T-cell responses in chronic HBV patients such that a functional cure could be achieved within a definitive treatment period.

Since HBV is a manageable disease, giving HBV carriers such combination therapy should raise ethical considerations. In our view, the most important argument for using the SIT in combination with low-dose combined ICI therapy in CHB patients is that NA treatment does not eliminate the risk of HCC, and thus 80 million people may die from liver cancer [2]. In this context, it is important to note that in a randomized clinical trial [18], nivolumab plus ipilimumab had manageable safety, a promising objective response rate, and durable responses even when registered doses were used (nivolumab 1 mg/kg plus ipilimumab 3 mg/kg) in patients with advanced hepatocellular carcinoma previously treated with sorafenib. Not unexpectedly, this protocol received accelerated approval in the US [19]. Such results are encouraging for the predicted safety of our ICI combination therapy in CHB patients, which administers only 0.5 mg/kg nivolumab plus 0.3 mg/kg ipilimumab [6,16].

**Figure 2:**
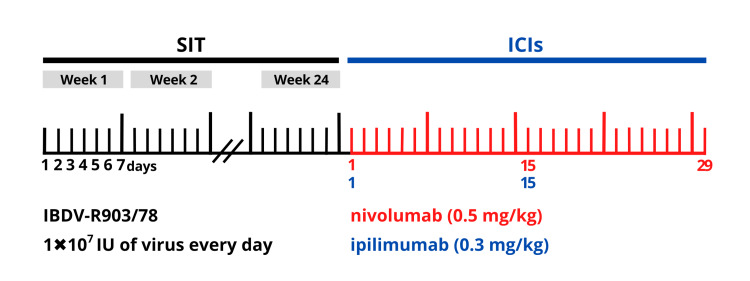
The planned combination of IBDV and ICI treatment courses for CHB patients The IBDV-R903/78 virus drug candidate (1 × 10^7^ IU/dose/day) will be orally administered in HBeAg-negative CHB patients daily for 24 weeks. Then, patients will intravenously receive nivolumab (0.5 mg/kg) on days 1, 15, and 29 (in red) and ipilimumab (0.3 mg/kg) on days 1 and 15 (in blue).

The new viral drug candidate R903/78 virus containing 1 × 10^7^ Infectious Units (IU) of IBDV will be administered orally in virally suppressed HBeAg-negative patients. The patients will be treated with the investigational product daily for 24 weeks. Then, off-label low doses of nivolumab (0.5 mg/kg) plus ipilimumab (0.3 mg/kg) will be administered [6] in order to restore exhausted HBV-specific T cells. Patients will receive nivolumab intravenously over 60 minutes on days 1, 15, and 29 and ipilimumab intravenously over 90 minutes on days 1 and 15 (Figure 2).

Blockading both coinhibitory receptors, the cytotoxic T lymphocyte-associated antigen-4 (CTLA‐4) and programmed cell death protein 1 (PD‐1) receptors, is required as they have nonredundant complementary roles in the attempt to recover an effective HBV‐specific T-cell response [16]. The primary objective of the proposed trial is to determine the safety profile of the R903/78 product used for the elimination of HBV infection in patients with CHB. The secondary objectives are to determine the efficacy of the R903/78 product in sequential combination with off-label low-dose ICI drugs in eliminating HBV infection in patients with CHB; to determine the effect of R903/78 on each of the following factors: (i) quantitative value of HbsAg, (ii) circulating HBV DNA, (iii) HBV RNA, (iv) HBV specific CD8 T cells, and (v) serum transaminase levels; to determine the safety of R903/78 and low doses of nivolumab and ipilimumab by assessing adverse events and laboratory parameters.

We predict that this new combination immune therapy will be safe, effective, and affordable as required in low-income countries where the burden of HBV infection is the highest [2]. Our testable hypothesis is based on the aforementioned information that SIT in combination with sequential low-dose nivolumab plus ipilimumab therapy is likely to provide synergistic activation of the immune system toward induction of therapeutic innate and adaptive anti-HBV responses aiming to accomplish functional cure in CHB patients during a finite treatment course. It is tempting to speculate that complementing our low-dose ICI protocol with a low-dose cyclophosphamide treatment in order to control regulatory T cells [20] would further improve the synergy between the protocol components. Therefore, we hope that this technical report will stimulate a meaningful discussion in the HBV research field.

## Conclusions

Based on the recommendation of the ICE-HBV, a sequential combination of two clinically validated modalities is proposed to achieve a functional cure of CHB patients. HBV replication will be suppressed by the broad-spectrum post-infection antiviral IBDV superinfection therapy. Exhausted HBV-specific T-cell responses will be restored by blockading the CTLA‐4 and PD‐1 receptors, respectively, with off-label low-doses of ipilimumab and nivolumab. IBDV activates the innate immune response by inducing IFN-stimulated genes from within, while ICIs restore the natural HBV-specific T cell response. Therefore, this combination therapy mimics the spontaneous resolution of HBV infection. The proof-of-principle of the new therapy will be demonstrated in virally suppressed HBeAg-negative patients.
